# Why Conscience Matters: A Theory of Conscience and Its Relevance to Conscientious Objection in Medicine

**DOI:** 10.1007/s11158-022-09555-2

**Published:** 2022-06-24

**Authors:** Xavier Symons

**Affiliations:** Plunkett Centre for Ethics, Australian Catholic University, 7 Ice Street, Darlinghurst, NSW 2010 Australia

**Keywords:** Moral psychology, Harm, Ground projects, Emotion, Integrity, Agency, Professionalism

## Abstract

Conscience is an idea that has significant currency in liberal democratic societies. Yet contemporary moral philosophical scholarship on conscience is surprisingly sparse. This paper seeks to offer a rigorous philosophical account of the role of conscience in moral life with a view to informing debates about the ethics of conscientious objection in medicine. I argue that conscience is concerned with a commitment to moral integrity and that restrictions on freedom of conscience prevent agents from living a moral life. In section one I argue that conscience is a principle of moral awareness in rational agents, and that it yields an awareness of the personal nature of moral obligation. Conscience also monitors the coherence between an agent’s identity-conferring beliefs and intentions and their practical actions. In section two I consider how human beings are harmed when they are forced to violate their conscience. Restrictions on the exercise of conscience prevent people from living in accord with their own considered understanding of the requirements of morality and undermine one’s capacity for moral agency. This article concludes with a consideration of how a robust theory of conscience can inform our understanding of conscientious objection in medicine. I argue that it is in the interest of individual practitioners and the medical profession generally to foster moral agency among doctors. This provides a prima facie justification for permitting at least some kinds of conscientious objection.

## Introduction

Conscience is an idea that has a significant currency in moral and political discourse in liberal democratic societies. The *International Charter on Civil and Political Rights*—ratified by nations such as Australia, Canada and the United States—recognises a ‘right to freedom of thought, conscience and religion’ (United Nations General Assembly 1976, art. 18). This right is also enshrined in the European Union Charter of Fundamental Rights (European Commission 2000). Indeed, liberal democracy is premised on the conviction that people should be allowed to live and act in accord with their deeply held beliefs, provided that such beliefs do not advocate or condone harm to others. Freedom of conscience has broad-ranging implications for social and political life. In many political jurisdictions, politicians are entitled to vote based on their conscience (rather than along party lines) on socially divisive issues. This is sometimes called a *conscience vote* (Beard 2011). In healthcare, practitioners are often granted a limited right to conscience objection. Medical associations in the United States, the UK and Australia all recognise a qualified right to conscientious objection in medicine (American Medical Association 2022; General Medical Council 2022; Australian Medical Association 2019). This right, however, has come under increasing pressure in recent years (Charo 2005; Savulescu 2006; Schuklenk and Smalling 2017; McLeod 2020).

Surprisingly, recent moral philosophical literature on the notion of conscience is sparse (Brownlee 2012, p. 3). In general, it is only in areas where freedom of conscience has become a source of debate that scholars have turned their attention to conscience as a feature of human moral psychology. Extant scholarship tends to offer a reductionist view of conscience, equating it with deeply held beliefs or a person’s religious or ethical commitments. Savulescu, quoting Shakespeare, has described conscience as a ‘word that cowards use’, warning that it can be an ‘excuse for vice’ or ‘invoked to avoid one’s duty’ (Savulescu 2006, p. 294). Similarly, Schuklenk and Smalling have argued that conscience claims by professionals are ‘untestable’ and ‘arbitrary’, and that accommodating for conscience claims could have very serious real-world consequences for people wishing to access public services (Schuklenk and Smalling 2017, p. 236).

In this essay, I intend to lay the moral psychological groundwork for a defence of freedom of conscience in professional life and medical practice in particular.^1^ I contend that recent criticisms of conscientious objection in medicine and other professions have been driven in part by a failure to adequately grapple with the concept of conscience. Scholars either provide a decidedly superficial account of conscience or do not even discuss conscience at all. To this end, this essay offers a detailed account of the role of conscience in moral life and considers why restrictions on conscience do grave harm to moral agents. I then outline how this robust understanding of conscience gives us reason to respect conscientious objection in medicine. It is my hope that this essay will provide a stimulus for a more thorough academic engagement with the concept of conscience and its relevance to professional life.

Section One of this article offers an account of the role of conscience in human moral psychology. I reject the idea that conscience provides us with a source of intuitive moral knowledge. Instead, I argue that conscience provides us with an awareness of the personal nature of moral obligation. That is, conscience leads agents to view morality in relation to their own character and identity and leads them to commit to living up to the requirements of the moral life. In Section Two, I consider how human beings are harmed when their freedom of conscience is restricted. Some theorists note that violations of conscience can lead to severe emotional trauma. Yet I focus on the implications that the suppression of conscience has for one’s character and capacity for agency. In Section Three, I offer a justification for permitting conscientious objection in medicine. I argue that respect for conscientious objection in medicine is a natural corollary of a recognition of the moral significance of conscience and the cost of violating conscience.

## The Role of Conscience in Human Moral Psychology: A Source of Moral Awareness Rather than Intuitive Moral Knowledge^2^

Conscience plays an indispensable role in giving agents an awareness of their moral duties and facilitating moral reflection. This section will discuss how conscience gives moral agents an *existential awareness* of their moral obligations. I will critique the widely held view that conscience provides intuitive knowledge about the rightness and wrongness of actions, and will instead argue that conscience yields an awareness of the personal relevance of moral obligation. I also argue that conscience monitors the coherence between an agent’s self-identifying beliefs and intentions and their practical actions (D’Arcy 1961; Blustein 1993; Benjamin 1995; Velleman 2009; Wicclair 2011; McLeod 2020).

Before commencing our discussion of conscience, it is important to note that this section will not provide an exhaustive discussion of the history of the idea of conscience; there are many ancient and modern philosophers who discussed the idea of conscience who I will not mention. Rather, the following discussion focuses on those philosophers who have been the most notable proponents of an intuitionist account of conscience (an account of conscience that is quite common in the literature today and that is thus necessary to critique). It should suffice to say that there are several traditions of theorising on conscience that are not discussed in this essay as comprehensive discussion of the history of conscience is not possible here.

Several early modern philosophers argued that conscience is a source of moral knowledge and plays an integral role in giving agents epistemic access to moral facts (Shaftesbury 1999/1711; Hume 1975/1740; Rousseau 1921/1762). What we are referring to here is not just the role that conscience plays in motivating agents to deliberate about how they ought to act. Rather, the claim is that conscience also gives agents a grasp of the meaning and importance of morality. That is to say, conscience gives us knowledge of the rightness or wrongness of actions. Conscience, in this regard, is not only a stimulus for obtaining moral knowledge but is actually a source of moral knowledge itself.

The meaning of the term ‘moral knowledge’ is indeterminate, however, and we must clarify what kind of moral knowledge conscience can be said to provide. Conscience is sometimes portrayed as a homunculus that whispers ethical guidance in the ears of moral agents. The commonly used metaphor of conscience as a ‘voice’ is an example of this homuncular view of conscience (Schinkel 2007, pp. 117–121). It is tempting, based on this caricature, to suggest that conscience gives us an intuitive grasp of moral truth. One might be inclined to describe conscience as ‘a distinct mental faculty, an intuitive moral sense that determines the rightness and wrongness of actions’ (Sulmasy 2008, p. 136).^3^ Rousseau was a proponent of this view and described conscience as ‘an innate principle of justice and virtue, by which…we judge our own actions or those of others to be good or evil’ (Rousseau 1762, p. 253). The idea is that conscience provides us with a clear and unadulterated view of the requirements of virtue and justice. According to Rousseau, conscience frees us from ‘childish errors’ and the ‘prejudices of our upbringing’ and provides a ‘true guide’ for virtuous action (Rousseau 1762, pp. 252–253).

Related to this, someone might describe the process of ‘searching one’s conscience’ as an introspective means of obtaining knowledge about morality. The thought might be that we have many conflicting ideas that characterise our moral reasoning—ideas that are the product of cultural, religious or political conditioning—and that introspection or soul-searching is required to get at those intuitions that form the true content of morality. One might argue that this process of introspection gives us access to conscience and that conscience is a faculty for moral perception that we discover deep in our consciousness. Bishop Butler, for example, described conscience as a faculty of the mind that ‘pronounces determinately some actions to be in themselves evil, wrong, unjust’ (Butler 1950/1726: Sermon II). One might think that we discern moral truth by making ourselves more attentive to these pronouncements of conscience deep in our consciousness.

Yet the intuitionist account of conscience does not seem right as conscience can often lead people to adopt manifestly immoral beliefs. If conscience were a privileged source of moral knowledge—a special sensibility deep in our consciousness that gives us privileged insight into morality—then it would seem that all moral reasoning could be concluded with the recommendation that each moral agent should ‘follow their conscience’ (Ojakangas 2013). Rather than engaging in moral reflection and dialogue to determine how one should act in particular situations, one could just follow the quiet voice echoing in the depth of one’s heart and use this as a moral guide. Yet experience indicates that the inner voice of conscience can be seriously misguided. Someone might, for example, have racist intuitions and sincerely believe that some races are superior to others. Other agents might have discriminatory intuitions about matters of gender, religion, class and so on. Conscience defined as a source of moral intuition, in this respect, does not seem infallible nor even reliable. Rather, in some cases it reflects a perverse moral logic.

Even if conscience in and of itself is reliable, agents can still face practical problems in trying to discern what guidance conscience is providing to them. The voice of conscience could be drowned out or mimicked by other features of our moral psychology. As Hill (1998) writes:

Whether or not we believe that conscience itself is infallible, we must still acknowledge that we can make mistakes about whether what we take to be dictates of conscience are authentic. Wishful thinking, fear, childhood prejudices, and indoctrination in false ideologies can imitate or distort the voice of conscience, especially if we have dulled that voice by frequently disregarding it.

The issue, then, is not just that conscience can itself err and lead moral agents astray. Even if conscience in and of itself is reliable, agents can still face practical problems in trying to discern what guidance conscience is providing to them. There are many things that can obscure the voice of conscience. What we might think is the voice of conscience may in fact be some prejudice that we have acquired in childhood or from the cultural or professional milieu in which we are immersed. As such, it is unclear whether conscience, in the intuitionist sense, could function as a useful guide for moral action. Rather, it seems that the voice of conscience would often be muted by other influences that shape our moral psychology.

There are significant problems, therefore, in describing conscience as a source of intuitive knowledge about the rightness or wrongness of particular acts. I do not want to completely disregard this view of conscience, as I do think there is merit to the idea that one must form one’s conscience for it to function as a reliable source of moral knowledge (O’Shea 2018). We can educate our own moral sensibilities in ways that will conform them more with moral truth. We can, for example, interrogate our intuitions in the light of widely accepted political or religious principles (such as a commitment to human rights or to the common good) and thus ensure that our intuitions are reliable. This qualification might help to rescue the intuitionist view from some of the powerful objections that it faces.

Yet in the context of debates about conscientious objection, I do not think it wise to focus on the intuitionist account of conscience—at least insofar as we are trying to make the case for ensuring that conscience is respected in law and professional codes of conduct. The intuitionist account of conscience raises too many concerns about the fallibility of our own moral compass. If we want to make the case for respecting consciences, we should be wary of focusing on the substantive content of the beliefs that conscience generates.

Moral knowledge is not limited, however, to substantive knowledge about the rightness or wrongness of particular acts. Rather, it is my contention that conscience gives us meta-ethical knowledge of the requirements of morality. Specifically, conscience provides us with an existential awareness of our moral obligations over and above our grasp of substantive moral truths. An agent can have moral knowledge about how they ought to act in a particular situation without that knowledge engaging them at a personal level. An agent may, for example, believe that theft is wrong without believing that they should refrain from engaging in acts of petty theft or, in a slightly different context, without refraining from unlawfully using another person’s intellectual property. The proposition that ‘theft is wrong’ may not move them in any deep way. Several philosophers (Fuss 1964; Blustein 1993; Sulmasy 2008) have argued that conscience is precisely that feature of our moral psychology that gives us an existential knowledge of our moral duties. That is, their view is that conscience provides an agent with the concrete, existential conviction that they ought to carry out those kinds of actions that practical reason has deemed to be morally obligatory.^4^ As Fuss writes, ‘conscience affords one the ‘existential knowledge’ (more properly, the existential conviction) that he is under obligation to do what he knows to be right and to pursue what he judges to be good’ (1964, p. 116). Similarly, Blustein writes that ‘conscience indicates a particular way of seeing moral and other normative demands, a mode of consciousness in which prospective actions are viewed in relation to one’s self and character’ (Blustein 1993, p. 294).

Some points of clarification are in order here. First, what we are describing is not a substantive belief arising from practical reason, nor a process of inference from general to particular moral knowledge. I am not arguing for an alternative formulation of the view that conscience provides us with intuitive knowledge about how we ought to act. Rather, the knowledge arising from conscience would be better described as a felt conviction that ‘one must act in accordance with what he knows or believes’ (Fuss 1964, p. 116). Conscience is a form of meta-ethical knowledge about how one ought to respond to the moral insights provided by practical reason as well as the beliefs that constitute one’s identity. This is a different kind of knowledge from the substantive knowledge that people typically associate with conscience, though it is still vital for living a moral life.

Conscience, according to this view, gives agents a sense that they have a personal stake in pursuing a moral life. This existential awareness provided by conscience leads one to make a commitment to conscientiously observing the requirements of morality. Thus Sulmasy (2008, p. 138) writes:

[Conscience involves] a commitment to uphold one’s deepest self-identifying moral beliefs; a commitment to discern the moral features of particular cases as best one can, and to reason morally to the best of one’s ability; [...] a commitment to make decisions according to the best of one’s moral ability and to act upon what one discerns to be the morally right course of action.

Conscience gives agents an awareness that their flourishing as moral agents is tied up with living a moral life in a conscientious manner and seeking to discern the good in particular situations. Conscience leads agents to ‘assent to the truth that one should act morally’ (Sulmasy 2008, p. 138).

It is important to be clear that conscience—understood as a form of moral awareness—does not necessarily track true moral knowledge. Conscience can, in some cases, be led astray by practical reason (Anscombe 2005, pp. 238–241). It may be the case, for example, that a soldier forms the belief that the torture of innocent civilians is morally permissible. The soldier may feel a strong existential conviction, arising from conscience, that they are duty bound to carry out torture if they receive such an order from a commanding officer. Conscience, then, could be said to yield a conviction that the soldier should do what, in the last analysis, is morally impermissible. So we should resist the claim that conscience is an infallible moral guide. This is not to say that conscience itself goes awry—strictly speaking, it is practical reason that errs. But conscience is responsive to practical reason, and as such will be implicated in the errors of practical reason (Fuss 1964, p. 117).^5^

To be clear, one of the practical functions of conscience is to ensure that there is coherence between our deep beliefs and our actions. Conscience monitors the extent to which our practical actions cohere with those beliefs that form part of our moral identity. As Sulmasy (2008, p. 138) writes:

The activity of conscience is a meta-judgment that arises in particular moral deliberations. It is a judgment that a proposed act (or an act one has already accomplished) would violate one’s fundamental moral commitments, including, importantly, a fundamental moral commitment to act with understanding.

People can, of course, have different fundamental moral commitments. Conscience does not prescribe what sorts of commitments a person must have. But it does monitor the extent to which a person is living up to these commitments, whatever they may be. Conscience is an advocate, so to speak, for the deeply held, identifying-conferring beliefs that a person holds.

It is in this respect that conscience can be said to track moral integrity. The integrity view of conscience has been widely defended in recent literature on conscience (Benjamin 1995, p. 470; Childress 1979, p. 322; Blustein 1993, p. 300; Sulmasy 2008, p. 138; Lyons 2009, pp. 488–494). As stated earlier, proponents of this view argue that conscience is concerned with personal integrity understood in terms of inner, psychological unity. Inner unity is valuable, according to these authors, either because integrity contributes to our having a good life (Benjamin 1995, p. 470; Blustein 1993), or because unity and the desire to repair ‘inner division’ are admirable characteristics of persons (Blustein 1993, p. 297). Conscience is a feature of our moral psychology that is responsible for preserving unity between our deeply held beliefs and our practical actions. It makes us alert to signs of discord between our actions or thoughts and our deep moral commitments; and it inclines us to assuage such discord.

One might question, however, whether it is in fact necessary to posit a level of moral awareness over and above the moral imperatives arising from practical reason. One might argue that practical reason alone is sufficient to explain moral motivation, and that we need not posit the existence of conscience to explain why people are committed to living a moral life. Conscience, on this picture, is redundant, at least when it comes to explaining why people live morally upright lives. Alternatively, one might argue that it is moral virtue, not conscience, that makes us responsive to moral reasons. We might describe virtues as the underlying dispositions that make agents responsive to the right kinds of moral reasons (Audi and Murphy 2006; Cullity 2017). That is, we might think of virtues as dispositions to act in the right way based on the right moral reasons. If indeed moral and intellectual virtues dispose an agent to respond appropriately to practical reason, then one might argue that it is unnecessary to posit the existence of a moral sensibility such as conscience whereby we become responsive to the imperatives of practical reason.

The trouble with these alternative perspectives, however, is that they focus on our responsiveness to specific moral reasons rather than the existential conviction that one ought to live a moral life. Practical reason tells us what the good is, or what the correct course of action is, and virtue makes us responsive to the right kinds of moral reasons. But neither practical reason nor virtues explain why we are personally invested in morality or why agents commit to living a moral life. Practical reason may very well yield specific moral knowledge in the form of normative propositions about the world. But this does not give an agent the conviction that they ought to act on this knowledge. Virtue may make us responsive to the right moral reasons, but it does not explain why an agent makes a fundamental commitment to living a moral life. Conscience is precisely that aspect of our moral psychology that yields a personal sense of moral obligation. It is that feature of psychology whereby an agent recognises that he is ‘under obligation to do what he knows to be right and to pursue what he judges to be good’ (Fuss 1964, p. 116).

Indeed, this is one significant way that my account of conscience differs from that offered by Kimberley Brownlee in her 2012 book *Conscience and Conviction: The Case for Civil Disobedience*. Brownlee describes conscience as a principle of moral responsiveness in agents and she links the cultivation of conscience to the cultivation of practical wisdom, virtue and objective moral integrity. Yet it seems that at least some of the descriptions that Brownlee provides of conscience—such as when she describes conscience as ‘a set of practical moral skills’ (p. 52) and ‘a guide to good conduct’ (p. 54)—make conscience sound a lot like practical reason or virtue. This approach runs the risk of rendering conscience redundant in our explanation of human moral psychology. It is this accusation of redundancy that I wish to avoid by describing conscience as *meta-capacity* which makes an agent aware of his or her personal investment in living a moral life. Granted, Brownlee also describes conscience ‘as a sustained commitment to improving ourselves as moral beings’ (p. 55), which makes her account sound much more like a meta-capacity. But she also at times appears to conflate conscience with lower-order moral psychological functions and I am inclined to reject these aspects of her theory.

This should suffice for a discussion of the role of conscience in moral life. I do not want to overstate the originality of my account of conscience. Ultimately, the position I am advancing is a close cousin of the view advanced by Brownlee as well as Sulmasy and others. The more novel aspect of my argument lies not in the content of the theory of conscience that I wish to advance but rather my discussion of the moral harm that an agent incurs when they act contrary to conscience. It is to this topic that we now turn.

## Acting Against Conscience and Moral Harm

This article has as its theme conscience and conscientious objection. In this section I will explore the moral cost of acting contrary to one’s conscience. Some theorists offer a detailed description of the emotional sanctions that agents experience when they act contrary to their conscience (Childress 1979; Lyons 2009). In this section, however, I will focus on the cost of acting against one’s conscience and the implications that this has for one’s self and one’s character. Indeed, it seems that we ought to focus on the self first and emotions second, at least insofar as we are to get to the heart of why conscience matters. I will argue that moral agents risk losing their basic orienting ideals should they act in a manner contrary to their deep moral and other normative commitments. Specifically, they imperil those life projects that are the very conditions for their existence and that give meaning and purpose to their lives. Agents who abandon their deeply held beliefs and commitments, furthermore, also undermine their capacity for independent moral judgement. Our personal moral ideals are an epistemic standpoint from which we can independently judge the social and professional norms of the communities to which we belong. To the extent that one lacks personal moral ideals, or one allows these ideals to be eroded, one loses the vantage-point from which one can independently critique the norms of professional work and social life.

Some theorists appeal to the notion of inner harmony or psychological integrity when attempting to describe the disvalue of acting contrary to conscience. In the previous section I described this as the integrity view of conscience. Wicclair, for example, refers to the importance of preserving moral integrity, which he defines as consistency between one’s actions and one’s core moral convictions (Wicclair 2017, pp. 7–8). Similarly, Childress (1979, p. 318) associates violations of conscience with a ‘fundamental loss of integrity, wholeness, and harmony in the self’. The question remains, however, as to why a loss of inner harmony or psychological integrity is of disvalue. Why is it problematic that we act in a manner contrary to our deep beliefs?

To answer this question, we should recall that conscience provides agents with a personal sense of moral obligation. It helps agents to see the requirements of morality in relation to their own self and character. Importantly, what we are focused on here is not an abstract conception of morality—such as a Kantian deontological framework or a utilitarian framework—to which an agent is bound by the force of reason. Rather, what we are concerned with is an agent’s own considered understanding of morality formed and sifted through the filter of their own life experiences (Williams 1981). What conscience draws an agent’s attention to is their own way of conceptualising the moral life and their own deep beliefs about their social and professional responsibilities. These beliefs may contingently overlap with a particular universalist conception of moral obligation (be it a Kantian deontological conception of morality, a utilitarian moral theory, or some other moral framework). Yet there is no necessary connection between any one moral framework and how an individual moral agent understands morality. Ultimately an agent’s conception of morality can be as subtly varied as the variety of human experience itself. What makes an agent’s conception of morality normative from the perspective of conscience is that it constitutes a fundamental part of her character and identity. By virtue of her conscience, an agent feels that she is bound—on pain of inauthenticity—to abide by the requirements of morality as she so conceives of it. Besides, conscience also consists of someone’s considered moral judgements about the world, and it makes sense for an agent to act in accord with their best judgement about the right course of action.

As I mentioned earlier, I do not claim that conscience never goes awry. Conscience can be misled by practical reason, or, alternatively, an agent may internalise a conception of morality that is, in the last analysis, fundamentally misguided. For example, it may be that an agent has internalised cultural norms that are manifestly misogynistic or even racist. Conscience, in this respect, could end up enforcing beliefs that are morally reprehensible. But we should not jump from this fact to the conclusion that conscience consists of nothing more than a series of arbitrary likes and dislikes. Quite the contrary, for a belief or commitment to form part of an agent’s identity it must be something that she has reflectively endorsed and held for a sustained period. It must be sincerely felt and shape the agent’s very outlook on life. Nothing is further from a whim than an agent’s deepest beliefs and commitments.

Here we arrive at a bedrock insight concerning the moral harm arising when one acts against one’s conscience. Acting against conscience does not only lead to emotional distress. The moral psychological reality is more profound than this. Agents also experience a weakened sense or total loss of meaning and identity when they transgress their deepest commitments. Bernard Williams (1981, p. 13) offers an insightful discussion of this matter, describing an agent’s basic commitments or ‘ground projects’ as ‘the motive force which propels him into the future, and gives him a reason for living’. Williams writes:

[it need not be the case] that if [an agent’s ground project] were frustrated or in any of various ways he lost it, he would have to commit suicide, nor does he have to think that… but he may feel in those circumstances that he might as well have died...in general a man does not have one separable project which plays this ground role: rather, there is a nexus of projects, related to his conditions of life, and it would be the loss of all or most of them that would remove meaning. (Ibid.)

My claim is that deep and repeated violations of conscience lead an agent to experience a loss of purpose and meaning and a concomitant loss of identity. The cost of acting against one’s conscience is higher where an agent acts contrary to not just one commitment but the very nexus of commitments that makes her life worthwhile. Self-betrayal of this kind would fall into the category of actions that are, from the perspective of an agent, ‘unthinkable’. I am referring to actions so contrary to an agent’s beliefs that he ‘cannot find anything in his self-conception to make it intelligible as something that he would do’ (Velleman 2009, p. 108). To seriously violate conscience is, in a very meaningful sense of the word, to do violence to one’s identity.

To use one example, we can consider the character of Sir Thomas More in Robert Bolt’s stage play *A Man for All Seasons*. The play focuses on the life and death of More, a Chancellor of England in the sixteenth century who famously refused to endorse Henry VIII’s decision to divorce his wife Catherine of Aragon. More was executed for this. At one point in the play, in a tense theological conversation with his friend the Duke of Norfolk, More defends his commitment to the Catholic conception of the indissolubility of marriage, stating: ‘what matters is not that it’s true, but that I believe it; or no, not that I believe it, but that *I* believe it’ (Bolt 1960, p. 110). More is here emphasising the fact that the belief is part of his identity, and that he feels bound in conscience to act in accord with the belief. More acknowledges that there may be other views about the permissibility of divorce that fall within the pale of reasonableness. But that is beside the point. He is committed to the Catholic conception, and therefore is bound in conscience to act in accord with this belief. To do otherwise would be to betray himself and to do violence to the ‘*I*’ that is the subject of the belief.

Suffice to say that conscience involves a commitment to acting in accord with one’s deep beliefs, and that a failure to do so can result in a dissolution of one’s own understanding of one’s identity (something that More believes is a moral harm worse than death).

The literature on moral injury is also a useful point of reference when trying to understand the psychology of conscience. Moral injury refers to the strong cognitive and emotional response that can occur following events that violate a person’s moral or ethical code. Potentially morally injurious events include a person’s own or other people’s acts of omission or commission, or betrayal by a trusted person in a high-stakes situation (Williamson et al. 2021). For example, healthcare staff working during the COVID-19 pandemic might experience moral injury because they perceive that they received inadequate protective equipment, or when their workload is such that they deliver care of a standard that falls well below what they would usually consider to be good enough (Williamson et al. 2021).

Moral injury is a much broader concept that has relevance beyond the psychology of conscience. Moral injury can be caused by the actions of others, such as when a trusted friend betrays you, whereas acting against conscience is an act of *self*-betrayal. Moral injury is thus a concept that ranges beyond betrayals of one’s conscience. But acting against one’s conscience is, nevertheless, a potentially morally injurious event and can produce the same effects as those described in the moral injury literature.

Specifically, violations of one’s conscience can produce a loss of meaning and moral identity akin to that experienced by the morally injured. Fontana and Rosenheck (2004) found that potentially morally injurious events in war (such as killing, enjoying killing, participating in atrocities, contributing to another’s death and failure to save wounded) positively predicted guilt, the experience of spiritual crisis and a loss of meaning in life. In some studies loss of meaning is deemed to be one of the most common experiences of people with moral injury (Ames et al. 2019). Some of the language used to describe moral injury also speaks to the idea of a loss of moral identity. Sherman describes moral injury as ‘global feeling of a sense of shattered moral identity, moral despair and or profound moral disillusionment’ (Sherman 2017, p. 1). Other authors use terms such as ‘moral affront’, ‘moral disruption’ (Drescher et al. 2011); ‘moral dislocation’ (Sherman 2015) and ‘moral disorientation’ (Molendijk 2018) to capture the notion of a loss of moral identity. These terms acknowledge the disturbance to one’s sense of self and character that moral injury can produce.

Something similar is liable to occur when someone violates their own conscience in a fundamental way. After all, violations of conscience undercut the basic beliefs around which one orients oneself in the morally complex world in which we live. Without such beliefs to orient oneself, it stands to reason that moral agents will experience moral disruption, dislocation and disorientation.

So much for the harm of violations of conscience to one’s sense of meaning and identity. Second, I would like to discuss the impact of acting against conscience on an agent’s capacity for moral agency. It is important to reflect on the criteria according to which we ascribe moral agency to an individual. I would like to focus on two related aspects of agency in particular. First, I would like to focus on the notion of a discretionary space in which a moral agent can make moral decisions in an unconstrained way. Second, I would like to focus on an agent’s reflective endorsement of those desires that motivate action. Both of these elements of agency are undermined when one acts against conscience in response to duress from an external authority. By a ‘restriction on conscience’, I will be referring to a conduct rule issued by an external authority that prevents an agent from acting in accord with her conscience.

First, we should recognise that moral agency requires discretionary space in which an agent is free to make their own moral decisions. It may sound like a truism to say that one requires freedom to make free decisions; yet there seems to be widespread confusion about this in the context of social and professional ethics. Some commentators, for example, believe that it is acceptable to enforce professional standards such that a health professional has no option of dissenting from mainstream practice (cf. Stahl and Emanuel 2017). Without the ‘discretionary space’ to choose between different options, however, a moral agent’s ‘choice’ of a particular action can only be said to be free in a highly qualified way (cf. Sulmasy 2017). It matters if the moral agent could have chosen otherwise. If someone’s actions were constrained such that she only really had one viable option from which to choose, then she can hardly be said to exercise moral agency in choosing this option. Rather, she would say that her agency has been diminished or distinguished by the constraints that have been imposed on her.

Second—and even if we reject the claim that agency requires that an agent has an ability to do otherwise—a moral agent’s capacity for agency is, at the very least, conditional on her reflective endorsement of the reasons and desires that motivate her actions. That is to say, for an agent to exercise moral agency, she must reflectively endorse the reasons and desires that lead her to act in particular ways. The agent must have pro-attitudes towards the reasons and desires in question, and must desire at a second-order level that the first-order reasons and desires that motivate them actually form part of their will. A failure to do this means that the agent remains ‘wanton’ or indifferent towards the reasons that drive their actions (cf. Frankfurt 1971). This is hardly an example of rational and reflective moral agency.

The trouble with a restriction on the exercise of conscience, however, is that it involves agents acting on the basis of coercion or compulsion rather than reflectively endorsed desires. If we force people to behave in particular ways, we are not allowing them to act based on reasons and desires that they have reflectively endorsed. Rather, we are leading them to act on the basis of duress, and there is a very real sense in which they are not exercising their agency—at least, not in the fullest sense of the word. For example, if someone commits a crime in the heat of passion, there is a sense in which her responsibility for that crime is diminished. She has not fully reflectively endorsed their course of action, and so cannot be said to be acting with the full force of her character. I would argue that something analogous is occurring when social or professional norms are enforced in such a way that individual moral agents have no choice but to conform to these norms. Agents’ adherence to these norms is motivated by an external force rather than by a rationally endorsed, interior conviction that one ought to act in accord with these norms.

It is instructive here to return to the idea of integrity, and to consider how this might be related to agency. Part of what it means to be a moral agent is to form an identity, based on one’s considered views of the world. We might think of this as an extended process of reflective endorsement, whereby one steadily acquires a series of identity-conferring beliefs that come to define her character. These beliefs and commitments, in turn, function as the content of one’s moral agency. MacIntyre (1999, p. 317) links integrity to maintenance of a fixed identity, and suggests that maintenance of one’s identity underpins one’s capacity for agency. He writes:

To have integrity is to refuse to be, to have educated oneself so that one is no longer able to be, one kind of person in one social context, while quite another in other contexts. It is to have set inflexible limits to one’s adaptability to the roles that one may be called upon to play.

Integrity, in other words, is precisely about not adapting to community practices that conflict with one’s moral code. If one were to be limitlessly open to adaptation based on social context, then one’s values would ultimately be a mere reflection of social context rather than reflectively endorsed commitments.

Some theorists may argue that adaptability is a virtue, particularly when one is discharging an important social or professional role. That is to say, it could be argued that it is virtuous to make oneself amenable to the relevant conventions that one encounters in one’s social or professional life. I would argue, however, that adaptability is only a virtue when one has manifestly fallen into moral error. In contrast, where one is indeed convinced upon reflection that one has arrived at the correct moral conviction vis-a-vis one’s social or professional obligations, it is a virtue rather than a vice to hold to one’s beliefs rather than adapting to the demands that have been placed on oneself by one’s peers. This is precisely what it means to have integrity. Conscience, for its part, is that aspect of our psychology that leads us to maintain integrity and to persist in our beliefs and commitments when these conflict with prevailing social or professional norms.

Agency follows on from integrity, as agency is about acting in a manner consonant with one’s desires. And those desires that are most truly our own are those that have been sifted through reflection and experience and that we have interiorised to form part of our character. We exercise moral agency, in the fullest sense of the word, when we act wholeheartedly (Frankfurt 1988). This wholeheartedness in turn requires at least some degree of inflexibility (enough inflexibility for us to maintain some grip on our own personal identity).

As I suggested earlier, agents who abandon their deeply held beliefs also undermine their capacity for independent moral judgement. Building on the foregoing discussion, we can say that our personal moral ideals are an epistemic standpoint from which we can independently judge the professional norms and standards that are imposed on us. They are a means by which we can step outside of our professional role and view the world from our own unique moral point of view, which may have been developed on the basis of (or may have been informed by) various moral frameworks or principles. By setting ‘inflexible limits’ on the kinds of social and professional functions that we are willing to perform, we maintain an important degree of epistemic and volitional independence from the social and professional communities of which we form a part. To the extent that we lack personal moral ideals, however—or, to the extent that we allow these ideals to be eroded—we lose the vantage-point from which we can independently critique the norms that characterise our social and professional communities. If the moral content of our character becomes indistinguishable from the moral conventions of these communities—or, perhaps more to the point, if we fail to sift these conventions through the filter of our own capacity for critical reflection—we lose the capacity for impartial and detached judgement that is necessary to externally critique social or professional conventions. If we are endlessly willing to adapt ourselves to any convention that is foist upon us, we will lose the agential independence necessary to externally critique the communities of practice of which we form a part.

A problem with restrictions on conscience, then, is that they undermine those features of moral rationality that are prerequisite of agency and independent judgement. Agents are encouraged to suppress and abandon those beliefs that ultimately form the bedrock of their unique, personal moral point of view. Rather, they are led to make themselves fully beholden to professional moral standards instead of following their own interiorised standards of what is right and wrong. These factors combine to undermine an agent’s capacity for independent moral judgement (which presumably is a core feature of moral agency). Restrictions on conscience, in this sense, greatly diminish an agent’s capacity for considered moral judgement and action independent of the norms of social and professional practices.

## An Application of Principles of Conscience to Conscientious Objection in Medicine

In this section I will consider how the preceding discussion of conscience can inform our understanding of the ethics of conscientious objection in medical practice. I have argued that violations of conscience gravely harm one’s sense of identity and one’s capacity for moral agency. If we accept this, then it seems that we ought to afford a prima facie right to conscientious objection to healthcare practitioners.^6^ Indeed, I would argue that the medical profession has an interest in cultivating practitioners of conscience who will in turn maintain and, where necessary, *critique* the moral standards in the profession. Without practitioners who morally reflect on their work and on the norms that characterise the profession, the institution of medicine would in a sense be left without a ‘voice of conscience’ that could call it to account when it errs. In what follows I will argue that there is a prima facie justification for permitting conscientious objection in medicine, notwithstanding the need to respect patient welfare and autonomy.

A practitioner’s integrity and capacity for moral agency may be harmed if they are obliged to participate in practices to which they have a deeply held moral or religious objection. As I stated earlier, violations of conscience are concerned with the weakened sense or total loss of self and character that an agent experiences when she transgresses her deepest (moral) commitments. At worse, they lead to this fundamental loss of meaning and purpose. When an agent not only violates some particular moral stricture but transgresses her deepest moral beliefs, she is liable to experience a loss of purpose and meaning. That is to say, the impact of a violation of conscience is more serious where an agent violates not just one commitment but the very nexus of commitments that makes their life worthwhile.

The issue with practices such as abortion and euthanasia in medicine is that they concern some of the most fundamental moral and religious beliefs that an agent has about human life (assuming, for argument’s sake, that the agent is a conscientious objector). The kind of moral harm involved concerns not just one belief but the very nexus of beliefs that characterise one’s moral identity and that make one’s life worthwhile. One’s beliefs about the value of human life are related to someone’s view of humanity and their understanding of human dignity. To violate these beliefs is indeed to imperil one’s very source of moral orientation in life. One is liable to experience deep moral distress if one is coerced into violating such beliefs.

On this point, it is instructive to review the social psychological research that has been conducted on conscientious objectors in healthcare. Debra Hanna, a researcher and registered nurse, has written about the visceral pain and cognitive dissonance experienced by nurses forced to participate in elective abortions (Hanna 2005). Hanna conducted a survey with ten nurses who had a moral objection to abortion but who had participated in the procedure. One of the nurses described the ‘gut-wrenching’ experience of violating one of her deep moral commitments:

I think the initial distress was that I was doing the wrong thing. That ending a life was doing the wrong thing, and that was the most gut-wrenching for me.

Another participant described a similar experience of distress:

I just burst into tears… It just felt like someone socked me in the gut, and I just thought, ‘Oh, I can’t believe this!’.

From these excerpts one gains a sense of how moral disorienting it is for healthcare practitioners to violate their fundamental commitments. Violation of conscience can have serious psychological and existential ramifications for clinicians. While Hanna’s is just one paper in an extensive and varied literature on moral distress in healthcare, it is nevertheless a reason to be cautious of attempts to downplay the impact of restrictions on conscientious objection.

We should also consider how restrictions on conscientious objection in medicine impact on the capacity for moral agency of medical practitioners. As I stated earlier, one major concern with restrictions on conscience is that they involve agents acting on the basis of coercion or compulsion rather than reflectively endorsed desires. If we force people to behave in particular ways, we are not allowing them to act based on reasons and desires that they have reflectively endorsed. Rather, we are leading them to act on the basis of duress, and there is a very real sense in which they are not exercising their agency—at least, not in the fullest sense of the word. This is not a good state of affairs for the profession of medicine, which ostensibly encourages its members to be morally reflective and conscientious practitioners.

The issue appears most acute when we consider our earlier remark about an agent’s capacity to externally critique the norms of medicine. Personal moral ideals are an epistemic standpoint from which one can independently judge the professional norms and standards that are received from communities of practice. They are a means by which one can step outside of one’s professional role and view the world from one’s own unique moral point of view (a perspective that has been developed over the course of one’s lifetime through moral reflection and that constitutes a unique perspective of the world). To the extent that one lacks personal moral ideals, however—or, to the extent that one allows these ideals to be eroded—one loses the vantage-point from which one can independently critique the norms that characterise social and professional communities. If the moral content of a doctor’s character becomes indistinguishable from the moral conventions of medical community—or, perhaps more to the point, if individual clinicians fail to sift these conventions through the filter of their own capacity for critical reflection—clinicians lose the capacity for impartial and detached judgement that is necessary to externally critique the prevailing norms of medicine.

As is well known, there have been many dark chapters in the history of medicine where unethical practices had become endemic to the profession and for many years went unchallenged by individual medical practitioners. The American Medical Association, for example, indirectly impeded African Americans from access to basic healthcare for over a century by deliberately blocking doctors of colour from entering the medical profession (Baker 2014). The medical profession also once sanctioned eugenics and classified homosexuality as a disease (Stahl and Emanuel 2017, p. 1382).

To avoid a repeat of these episodes, it would seem advisable to cultivate a keen capacity for independent moral judgement in current practitioners in the field. Conscientious objection functions as an important bulwark against these aberrations in medicine. In the late 19th and early 20th centuries, for example, there were a small number of American clinicians who, in addition to religious leaders and politicians, spoke out about eugenic practices such as forced sterilisation and other forms of birth control. This resistance—in addition to other factors such as the horrors of WWII Nazi eugenics—contributed to the American Medical Association’s gradual change of position on eugenic policies and practices (Rosen 2004).

We undermine the good work of conscientious objectors, however, when we restrict a medical practitioner’s ability to exercise their conscience in their professional work. There is a prima facie argument, therefore, for permitting medical and other healthcare practitioners to opt out of practices that they believe to be immoral. While professional associations in medicine strive to ensure that doctors practise in an ethical manner, history shows that sometimes the majority of practitioners can be misguided on particular moral questions. It is part of a historically informed moral humility that we permit conscientious objection in medicine today.

In practice, a right to conscientious objection would mean that health authorities and senior clinicians should respect the deeply held beliefs of those practitioners under their jurisdiction. Appeals to conscience should be respected provided there is no risk of serious harm to patients. With Sulmasy I would contend that conscientious objection should be permitted provided it is not ‘destructive to society’ in one way or another or that poses a ‘risk of serious illness, injury, or death’ to a patient (Sulmasy 2017, p. 28). Exceptions would occur when a doctor’s conscientious objection is manifestly unreasonable and cuts against the basic principles of decency that underpin liberal democracies.

These remarks are, however, cursory and not intended to be an exhaustive defence of conscientious objection in medicine. One could, of course, argue that patients have an equal if not stronger claim to doctors on having their consciences respected. Publicly available services such as abortion are necessary to support individual capacities for moral agency. Without these services, a woman would be unable to exercise their autonomy vis-a-vis their own reproductive lives. Patients, after all, find themselves in a position of considerable vulnerability in the medical system, and are dependent on doctors to have their health needs met. A female patient who is unable to terminate an unwanted pregnancy, for example, is limited in her ability to pursue certain career and lifestyle options and, worse, may be at risk of psychological illness because of the pregnancy. It could be argued that respect for conscience requires that a doctor assist the patient in obtaining an abortion.

This is a counterargument that I will not seek to refute in detail, if only because I am not attempting to offer an exhaustive defence of the permissibility of conscientious objection in medicine. I would, however, note that it is not typically the case that a doctor’s conscientious objection would categorically prevent a patient from accessing a service that they sought. Indeed, abortion is, for the most part, readily available in jurisdictions where the procedure is legally permitted (though scholars have highlighted some notable exceptions (Minerva 2015)). Insofar as a doctor’s conscientious objection did prevent a patient from obtaining the service they desired, then it seems that the above counterargument fails to undermine the case in favour of conscience rights.

In any case, my aim is simply to illustrate how a conception of conscience grounded in identity-conferring commitments and individual agency can provide support for the kind of appeals to conscience that sometimes arise in the context of medical practice. There is real moral harm to acting against one’s own conscience, and restrictions on conscientious objection can precipitate such moral harm for clinicians who hold a conscientious objection to certain medical procedures, notwithstanding countervailing considerations pertaining to patient welfare and rights.

## Conclusion

In this article, I have provided an account of the role that conscience plays in moral life. I argued that conscience is a principle of moral awareness in human beings, and that it gives agents an awareness of the personal relevance of moral obligation. I then offered an overview of the loss of character and purpose that agents experience when they violate their conscience. I argued that moral agents risk losing their basic orienting ideals if they act in a manner contrary to their deep moral and other normative commitments. Agents who abandon their deeply held beliefs, furthermore, also undermine their capacity for independent moral judgement. Our personal moral ideals are an epistemic standpoint from which we can independently judge the social and professional norms of the communities of practice to which we belong. To the extent that one lacks personal moral ideals, or one allows these ideals to be eroded, one loses the vantage-point from which one can independently critique the norms of professional work and social life. The final section of this article considered how this account of conscience might inform our attitude towards a doctor’s right to conscientious objection in medicine. We have a prima facie reason to permit conscientious objection in medicine, I argued, on account of the centrality of conscience to an agent’s sense of meaning and purpose in life as well as to ensure that moral aberrations in medical practice do not go unchallenged.

I do not pretend, however, to have provided a comprehensive defence of conscientious objection in medicine. Indeed, there is much more we could say about the moral psychology of conscience. Rather, I simply hope to have laid the argumentative groundwork for other scholars wishing to defend a robust account of conscience and the practice of conscientious objection in healthcare. It is my hope that this article will be the stimulus for a rigorous scholarly discussion of the role that conscience plays in professional life. While many scholars have been quick to criticise the practice of conscientious objection in professions such as medicine, there has been limited engagement with the notion of conscience and its relevance to evaluating appeals to conscience in different social and professional contexts. This article has argued that agents suffer significant moral harm if they are forced to violate their conscience (a harm that goes much deeper than mere emotional trauma). This should be taken into account when evaluating the moral permissibility of restrictions on conscientious objection or the exercise of freedom of conscience. It is high time that we give due attention to what is all too often dismissed as an insignificant feature of human moral psychology.

## Footnotes

1 To be clear, I will not provide such a defence myself. It is my hope, however, that this essay provides important background argumentation for a defence of conscientious objection in healthcare by other scholars.

2 In this section I will focus on what one might call a ‘well-developed conscience’. It is true that someone might have a conscience that has been poorly formed, depleted or emasculated such that they may deceive themselves about their actions/intentions/desires or be unaware of their responsibilities and obligations. For the most part, this article will be dealing with a conscience that is well-formed and well-functioning.

3 To be clear, Sulmasy (2008) rejects this view. His own view is much closer to my own.

4 It is apposite to clarify how I am using the term *practical reason*. My understanding of this capacity of the mind leans heavily in the direction of Aristotle and Aquinas. Practical reason, in my view, refers to the capacity of the human mind for reasoning and judgement about human action. It is concerned with how we ought to act and behave, rather than speculative or theoretical matters. Practical reason is responsible for applying the first principles of morality to particular real-world moral situations.

5 The judgements of practical reason, in other words, form part of the content of conscience. If practical reason errs, then conscience will err, too.

6 Medical doctors are often asked to engage in practices that they find distasteful or in some other way undesirable. Yet it is not medical practices that one finds ‘distasteful’ that we are concerned with here. Rather, what we are concerned with are those practices to which a doctor may have a moral or religious objection. We might, for example, think of therapeutic abortion or voluntary euthanasia, though our understanding should not be limited to these cases.
